# The Influence of Native Deer on Forest Fauna—A Systematic Map

**DOI:** 10.1002/ece3.70696

**Published:** 2024-12-23

**Authors:** Sebastian Schwegmann, Manisha Bhardwaj, Ilse Storch

**Affiliations:** ^1^ Chair of Wildlife Ecology and Management University of Freiburg Freiburg Germany

**Keywords:** abundance, cervids, community, fauna, forest, species richness

## Abstract

Deer are the most abundant large herbivores in temperate and boreal forests across the Northern Hemisphere. They are ecosystem engineers known to alter understory vegetation and future tree species composition by selective browsing. Also, deer have strong impacts on faunistic groups, often mediated by vegetation. The ongoing loss of faunal biodiversity in forests worldwide can be exacerbated by high deer population densities. Adapted deer management for the purpose of forest biodiversity conservation requires a holistic understanding of deer–fauna relationships. In this systematic map, we examine the existing literature assessing the effects of deer on faunal communities in boreal and temperate forests. Our aim is to synthesize currently described trends and identify research gaps for our understanding of deer as biotic drivers of forest communities. We reviewed 64 studies on how the abundance, species richness, or diversity of faunal taxa responded to different levels of deer abundance or density in forest ecosystems across the Northern Hemisphere. In total, we found almost 400 individual reported effects of nine native deer species on forest‐dwelling faunal communities. However, our systematic map reveals that comprehensive synthesis of the current literature remains a challenge. Published studies often do not report contextual data essential for comparison and meta‐analysis, for example, deer density, forest management, and predation pressure. Moreover, the methodological approaches of the included studies often only account for potential linear effects of deer on fauna, likely oversimplifying the complexity of direct and indirect effects that deer can have on their ecosystem. We recommend that multi‐level enclosure experiments be applied to assess the impact on faunal taxa. This approach combines robust causal inference with the potential straightforward comparison and replication between deer species, forest types, and system productivity, which will facilitate the utilization of the results in future research and management.

## Graphical abstract

Deer are key drivers of faunal communities in temperate and boreal forest ecosystems. In this systematic map, we review the current literature assessing the effects of native deer on abundance, species richness, and diversity of forest‐dwelling faunal taxa. While many aspects of deer–faunal relationships are already studied, proper meta‐analysis requires better reporting of contextual data such as deer density and the assessment of potential non‐linear effects of deer on faunal taxa.

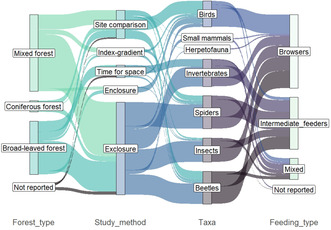

## 1 Introduction

Many forest‐dwelling animals are threatened with extinction throughout temperate and boreal biomes (Brockerhoff et al. 2017; Estes et al. 2011; Paillet et al. 2010). The primary causes of population decline include habitat deterioration resulting from human interventions, such as intensive timber production. While forestry and other human activities often affect forest specialists, the abundance and range of large herbivores such as deer (*Cervidae*) continue to increase across the Northern Hemisphere (Côté et al. 2004; Fuller 2001a; Valente et al. 2020). The removal of large predators, reduced competition from livestock, warmer winters, decreased hunting pressure, and favorable land use have resulted in deer population densities that are at their historic high (Côté et al. 2004; Fuller 2001a; Newson et al. 2012; Ripple et al. 2014).

Deer are often considered keystone species (Waller and Alverson 1997), that significantly shape their ecosystem including faunal and floral communities through herbivory (Boulanger et al. 2018; Chollet et al. 2016; Iida, Soga, Hiura, et al. 2016; Seki and Koganezawa 2013; Shen et al. 2016). At the same time, deer can also cause conflict with forest management due to their selective browsing, which results in biotic filtering of tree regeneration (Ammer 1996; Gill 1992, 2001; Hothorn and Müller 2010; Partl et al. 2002). Specifically, the current climate change‐related forest disturbances require management strategies that facilitate species‐rich tree regeneration for more resilient forest stands, which may be inhibited by selective deer browsing (Berthelot et al. 2021; Dymond et al. 2016; Jactel et al. 2017; Li et al. 2023). Generally, the extent of the impacts that deer have on the ecosystem can be attributed to their abundance. At low abundance, deer integrate into the ecosystem and their behavior has little lasting effect (Cordeiro Pereira et al. 2024; Gill and Morgan 2010; Hanberry and Faison 2023). At intermediate levels, the impact of deer populations can lead to an increase of plant and animal diversity, according to the “intermediate disturbance hypothesis” (Connell 1978; Miller, Roxburgh, and Shea 2011; Roxburgh, Shea, and Wilson 2004). While this hypothesis has been found not to be universally applicable (e.g., Fox 2013), it may still be applicable in the context of deer effects on forest ecosystems. Specifically in moderate abundances, deer have been demonstrated to increase species diversity, by fulfilling a range of ecosystem functions including seed dispersal (Iravani et al. 2011; Jaroszewicz, Pirożnikow, and Sondej 2013), delaying the closure of forest gaps that are important for many plant and animal species (Burton et al. 2021; Cardinal, Martin, and Côté 2012; Feber 2001; Hanberry and Faison 2023; Muscolo et al. 2014), and providing dung and carcasses for the necrophagous and coprophagous fauna (Buse et al. 2021; Iida, Soga, and Koike 2016; Schwegmann et al. 2022; Selva et al. 2005). At high abundances, deer are often considered “overabundant” (Caughely 1981; McShea, Underwood, and Rappole 1997), and may disrupt ecosystem functioning by limiting young tree growth, plant diversity, and negatively affecting forest dwelling faunal taxa (Côté et al. 2004; Crystal‐Ornelas et al. 2021; Wheatall, Nuttle, and Yerger 2013). The threshold of when deer populations are deemed “overabundant” is strongly context‐dependent and not clearly defined (Côté et al. 2004).

Overall, interactions between deer and forest vegetation have been intensively studied (Bernes et al. 2018; Takatsuki 2009), often with a focus on managed forest systems and woody vegetation, due to high their relevance for forest managers and/or recreational hunters. The consensus tends to be that high deer abundance negatively impacts elements of biodiversity such as tree regeneration, understory vegetation, and faunal taxa (Côté et al. 2004; Goetsch et al. 2011; Sakata and Yamasaki 2015; Stewart 2001). However, this view tends to be vegetation biased, specifically by focusing on browsing pressure on young trees, while disregarding other taxa and ecological functions of deer behavior (Goetsch et al. 2011; Jenkins et al. 2014; Schulze et al. 2014). Deer have widespread effects on faunal taxa as a result of vegetation changes due to browsing (Melis et al. 2006; Takada et al. 2008). Moreover, deer also affect soil characteristics, nutrient cycling, microclimate, and sedimentation patterns in adjacent streams with consequences for faunal assemblages (Mahon and Crist 2019; Mohr and Topp 2001; Nakagawa 2021; Prietzel and Ammer 2008; Shelton et al. 2014; Suominen 1999; White 2012).

Deer species can be categorized into two different feeding types: browsers and intermediate feeders. Browsers, or concentrate selectors, while adaptable, generally select for high quality forage with a high concentration in protein, often found in herbs or shoots of woody vegetation (Clauss et al. 2008; Hofmann 1989; König et al. 2020). Intermediate feeders, on the other hand, are more generalist and feed more on cellulose‐rich biomass such as grasses, while not being full grazers (Gebert and Verheyden‐Tixier 2001). Generally, herbivores can shift the floral community composition by selective feeding on specific plants which gain a competitive disadvantage, or giving species that are more able to compensate tissue removal a competitive advantage (e.g., graminoids vs. annuals) (Bernes et al. 2018; Hegland, Lilleeng, and Moe 2013). Furthermore, as the difference in foraging behavior moderates how deer affect the forest understory vegetation, it is likely that browsers and intermediate feeders also have different impacts on the forest fauna (Bernes et al. 2018; Faison et al. 2016; Hegland, Rydgren, and Goslee 2016).

Ongoing biodiversity loss worldwide seriously threatens the resilience of forest ecosystems (Dovčiak and Halpern 2010; Downing et al. 2012; Oliver et al. 2015). In addition to climate change and habitat fragmentation, forest managers and conservationists also need to understand on how biotic drivers such as large herbivores affect forest biodiversity, to prevent further biodiversity loss. Previous reviews in this field focus on specific faunal groups (Bernes et al. 2018; Feber 2001; Flowerdew 2001; Fuller 2001b; Phillips and Cristol 2024; Stewart 2001), while others include more herbivore taxa (Dolman and Wäber 2008; Katona and Coetsee 2019), specifically assess “overabundant” deer populations (Crystal‐Ornelas et al. 2021), or synthesize studies across a wider range of different habitat types (Foster, Barton, and Lindenmayer 2014; Suominen and Danell 2006). Previous synthesis attempts in this field did not assess the full range of biotic interactions between deer and forest fauna, and focussed on specific taxa or unusually deer abundances. The aim of this systematic map is to summarize the available literature on deer as drivers of forest faunal communities and to identify research gaps, in order to point the way toward future syntheses and a holistic understanding of deer–fauna relationships (James, Randall, and Haddaway 2016). In contrast to previous reviews, we focus on native deer species in temperate and boreal forest ecosystems. While introduced, herbivores can also be strong drivers of biotic communities, the effects of introduced herbivores are expected to be inherently different due to a lack of coevolution and adaptation, whereas native species are more likely to perform important ecosystem functions (Bernes et al. 2018).

## 2 Methods

We used a comprehensive and systematic approach to assess published studies on the effect of deer on faunal taxa using the search engine *Web of Science* with a search string that can be found in Appendix S1. The resulting studies were filtered in three steps: exclusion based on title, exclusion based on abstract, and finally exclusion based on full text. In general, we searched for empirical studies that assessed the response of faunal communities (abundance, species richness, and diversity) to varying deer abundance in forest ecosystems of the Northern Hemisphere. We used the following criteria for inclusion or exclusion of studies in the systematic map:

1. Empirical studies, including notes, assessing the effects of varying deer abundance or population density on the abundance, species richness or diversity of other faunal taxa. We excluded studies that only aimed to evaluate the relationship between deer and specific species. We included studies that directly or indirectly measured deer presence/absence, abundance or population density using methods such as fencing, site comparisons, time for space approaches or index methods (e.g., pellet counts) to generate deer‐related predictor variables. We excluded studies that focused on deer behavior, lacked information or evidence on deer abundance, or simulated deer behavior (e.g., branch clipping). For responding faunal taxa, we allowed biomass, activity or density as proxies for abundance;
2. The study was conducted in temperate or boreal forests in the Northern Hemisphere. We did not include studies that assessed the effects of deer on faunal communities in streams;
3. The *Cervidae* population studied was native to the study area or introduced more than 500 years ago. Thus, we included studies with the following deer species in their native range in forests of the Northern Hemisphere: red deer (*Cervus elaphus)*, elk (*Cervus canadensis*), sika deer (*Cervus nippon*), fallow deer (*Dama dama*), moose (*Alces alces*), roe deer (*Capreolus capreolus*), white‐tailed deer (*Odocoileus virginarius*), mule deer (*Odocoileus hemionus*), or reindeer (*Rangifer tarandus*). We did not exclude studies in which introduced deer, such as Reeves's muntjac ( *Muntiacus reevesi* ), occurred alongside native deer. Similarly, we only excluded studies on other herbivores such as moufflon (*Ovis gmelini*), when these, rather than deer, appeared to have the greatest impact on the responding taxa according to the authors;
4. Published in peer‐reviewed English‐language journals;
5. The most recent and/or with the largest dataset from multiple studies of the same taxa at the same study sites, unless the deer abundance in the study area changed over time, in which case we included multiple studies from the same study site focusing on the same response taxa.

In addition to the systematic search, we also included any studies referenced in the initial selection of studied but did not appear in the search, if they met the above criteria. After finalizing the set of included studies, we extracted information on the study sites, deer species studied, the responding faunal taxa and other methodological and contextual aspects of the study context. Overall, we followed the PRISMA guidelines for the preparation of this review (O'Dea et al. 2021).

For the study sites, we extracted the country of the study site and classified whether the study was conducted in a temperate or boreal ecoregion, either based on the study site description or based on Olson et al. (2001). We also classified whether the study was conducted in broad‐leaved, mixed, or coniferous forest based on the study description. If study sites were located in multiple forest types (e.g., coniferous and mixed stands), the study was fully assigned to the mixed forest category.

For each study, we classified which deer species were present and whether multiple deer species co‐occurred in the study area. Moreover, we extracted whether studies assessed the effects of only browsers, only intermediate feeders or of a mix of both feeding types. Among the species assessed in this study roe deer, white‐tailed deer, mule deer, and moose are classified as browsers (Hofmann 1989; Robbins, Spalinger, and van Hoven 1995; Tixier and Duncan 1996), while reindeer (red deer, elk, sika deer, and fallow deer are considered intermediate feeders (Hofmann 1989; Kalb, Bowman, and Deyoung 2018; Mcshea 2012; Ozaki et al. 2007).

For each study, we extracted which faunal groups were assessed and what effects the study reported. We aimed to report the effects of deer on faunal groups at the lowest taxonomic level possible, however our categorizations were driven by the studies themselves, and reflect categorizations that encompassed the most studies. We, therefore, grouped vertebrates into birds, herpetofauna, and small mammals. Effects on birds were frequently reported at the feeding or nesting guild level, therefore we also report these effects separately, as separating faunal groups into guilds may enhance our ecological understanding of the effects. Invertebrates were categorized as follows: invertebrates (in general), Annelida, Myriapoda, Crustacea, Gastropoda, spiders, other arachnids, and insects. Studies on spiders often reported the effects of deer specifically for either web‐building spiders or hunting spiders. Therefore, we also report the effects at this level and additionally classified effects reported for specific spider families into these groups using Cardoso et al. (2011). Insect studies were often order‐specific, particularly for Diptera, Hymenoptera, Lepidoptera, and Coleoptera. Coleoptera were also further classified as carabids, carrion, dung, or other. For all responding taxa, we assessed whether abundance, species richness, or diversity (using diversity indices such as the Shannon–Wiener index) was studied. If the response of faunal abundance to deer was only assessed at the species level, we counted this as only one effect for abundance. Additionally, we explored whether faunal community composition was studied at species level.

**FIGURE 1:**
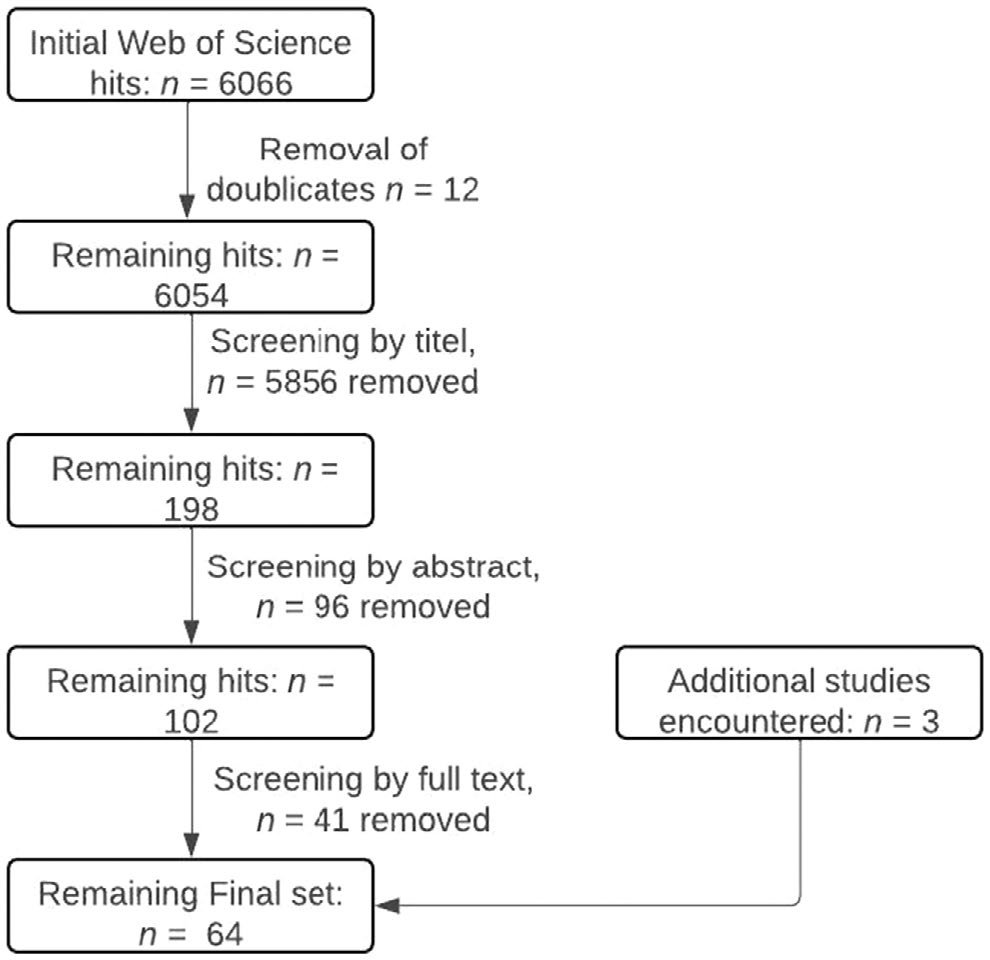
Flowchart of systematic literature screening process.

To assess the context in which the effects of deer on other fauna are studied, we assessed and extracted data on the methodological approach, the reported deer density, whether natural predators of deer were present in the study areas, whether the deer population was exposed to hunting in the study areas, and whether the forest was managed. Map and figures, with the exception of Figure 1, were generated using R (R Core Team 2021).

## 3 Results

### 3.1 Resulting Publications

**FIGURE 2:**
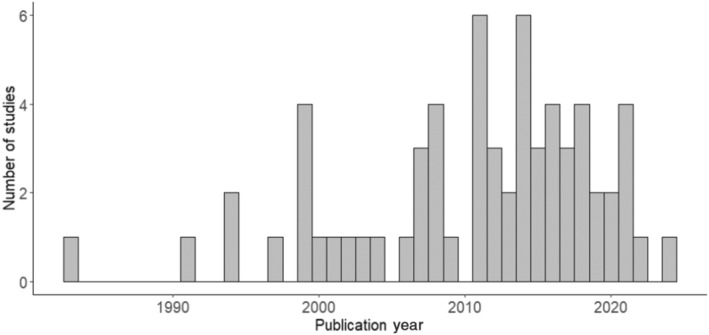
Histogram of the year of publication for the studies included into the review.

We conducted our systematic literature search on February 2, 2024 and did not impose any restriction on publication date. The systematic search started with a total of 6066 initial hits and 64 studies were finally included in the systematic map (Figure 1). More than half (65.6%) of the studies included in this review were published after 2010 (Figure 2).

**FIGURE 3:**
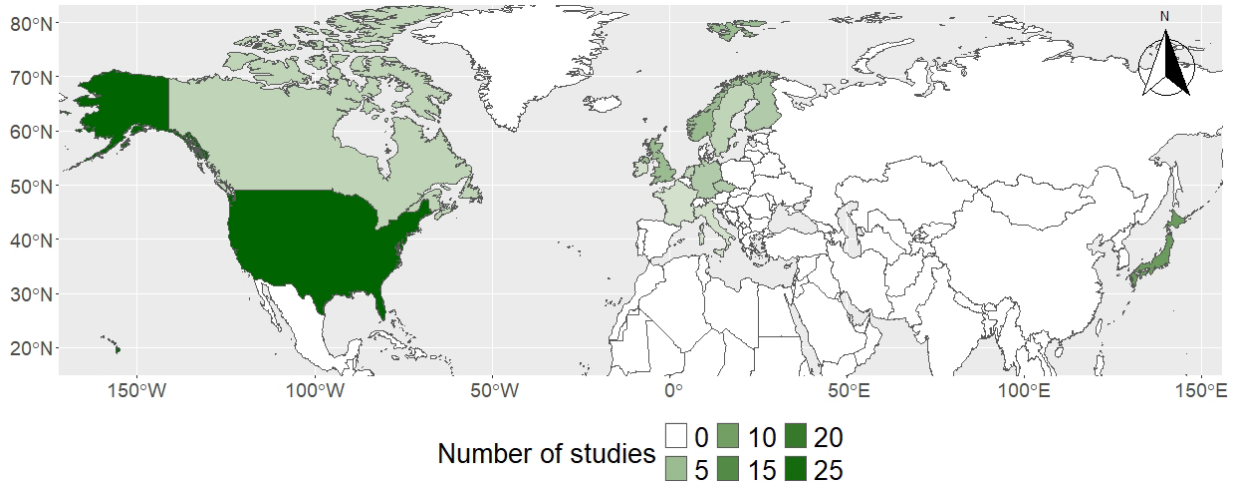
Map displaying the distribution of study locations of included studies in the Northern Hemisphere.

Of all included studies, most were conducted in North America (*n* = 29), while 24 and 11 studies were conducted Europe and Asia, respectively (Figure 3). In Asia, all included studies were exclusively conducted in Japan (*n* = 11). Overall, only 17.1% of the included studies (*n* = 11) were conducted in boreal forests. With regard to forest type, 14% of the studies were exclusively conducted in coniferous forest, 39% of the studies were conducted in pure deciduous forests and mixed forests, and 7.8% of the studies did not specify the forest type or were conducted at very large spatial scales. All studies included in the systematic map are listed in Table 1.

**TABLE 1:** List of studies included in the systematic map based on the inclusion and exclusion criteria.

Study	Deer species	Faunal taxa
Casey and Hein (1983)	Elk, white‐tailed deer	Birds
DeGraaf, Healy, and Brooks (1991)	White‐tailed deer	Birds
Baines, Sage, and Baines (1994)	Red deer	Invertebrates
deCalesta (1994)	White‐tailed deer	Birds
Roininen, Price, and Bryant (1997)	Moose	Insects
Suominen (1999)	Moose, reindeer, roe deer	Gastropods
Suominen, Danell, and Bergstrom (1999)	Moose, roe deer	Invertebrates
Suominen, Danell, and Bryant (1999)	Moose	Insects
Brooks (1999)	White‐tailed deer	Herpetofauna
McShea and Rappole (2000)	White‐tailed deer	Birds
Smit et al. (2001)	Red deer, roe deer	Mammals
Bailey and Whitham (2002)	Elk	Arthropods
Suominen et al. (2003)	Moose, reindeer	Beetles
Miyashita, Takada, and Shimazaki (2004)	Sika deer	Spiders
Benes et al. (2006)	Red deer, fallow deer	Lepidoptera
Gill and Fuller (2007)	Red deer, fallow deer, sika deer, roe deer, Muntjac	Birds
Melis et al. (2007)	Moose	Beetles
Kleintjes Neff, Fettig, and VanOverbeke (2007)	Elk	Lepidoptera
Takada et al. (2008)	Sika deer	Spiders
Greenwald, Petit, and Waite (2008)	White‐tailed deer	Invertebrates, herpetofauna
Spitzer et al. (2008)	Red deer, Fallow deer	Invertebrates
Saitoh et al. (2008)	Sika deer	Invertebrates
Huffman et al. (2009)	Elk, mule deer	Arthropods
Duguay and Farfaras (2011)	White‐tailed deer	Invertebrates
Mathisen and Skarpe (2011)	Moose	Birds
Martin, Arcese, and Scheerder (2011)	Mule deer	Birds
Nuttle et al. (2011)	White‐tailed deer	Lepidoptera, birds
Byman (2011)	White‐tailed deer	Mammals
Buesching et al. (2011)	Fallow deer, roe deer, muntjac	Mammals
Bressette, Beck, and Beauchamp (2012)	White‐tailed deer	Invertebrates
Christopher and Cameron (2012)	White‐tailed deer	Arthropods
Lessard et al. (2012)	NA	Arthropods
Tymkiw, Bowman, and Shriver (2013)	White‐tailed deer	Birds
Parsons, Maron, and Martin (2013)	Elk, mule deer, white‐tailed deer	Mammals
Suzuki and Ito (2014)	Sika deer	Invertebrates
Koike et al. (2014)	Sika deer	Beetles
Fuller et al. (2014)	Red deer, sika deer	Spiders
Holt, Fuller, and Dolman (2014)	Fallow deer, roe deer, muntaj	Birds
Pedersen et al. (2014)	Moose	Mammals
Shelton et al. (2014)	White‐tailed deer	Mammals, invertebrates, Herpetofauna
Chips et al. (2015)	White‐tailed deer	Insects
Katagiri and Hijii (2015)	Sika deer	Arthropods
G. Palmer et al. (2015)	Fallow deer, roe deer, muntajc	Birds
Roberson et al. (2016)	White‐tailed deer	Spiders
Baltzinger et al. (2016)	Red deer	Birds
Iida, Soga, Hiura, et al. (2016)	Sika deer	Beetles
Iida, Soga, and Koike (2016)	Sika deer	Beetles
Jirinec, Cristol, and Leu (2017)	White‐tailed deer	Birds
Landsman and Bowman (2017)	White‐tailed deer	Spiders, invertebrates
Katagiri and Hijii (2017)	Sika deer	Invertebrates
Lilleeng et al. (2018)	Red deer	Beetles
Machar, Cermak, and Pechanec (2018)	Fallow deer, roe deer	Birds
Gobbi et al. (2018)	Red deer	Beetles
Iida, Soga, and Koike (2018)	Sika deer	Beetles
Mahon, Campbell, and Crist (2019)	White‐tailed deer	Hymenoptera
Saikkonen et al. (2019)	Reindeer	Spiders
Taniwaki, Tamura, and Watanabe (2020)	Sika deer	Hymenoptera
Rushing et al. (2020)	White‐tailed deer	Birds
Bucher et al. (2021)	Red deer, roe deer	Spiders
Buse et al. (2021)	Red deer	Beetles
Lilleeng et al. (2021)	Red deer	Lepidoptera
Ramirez et al. (2021)	Red deer, fallow deer, roe deer	Mammals, invertebrates
Beckett et al. (2022)	Mule deer	Hymenoptera
Cordeiro Pereira et al. (2024)	Roe deer	Beetles

### 3.2 Focal Deer Species

**FIGURE 4:**
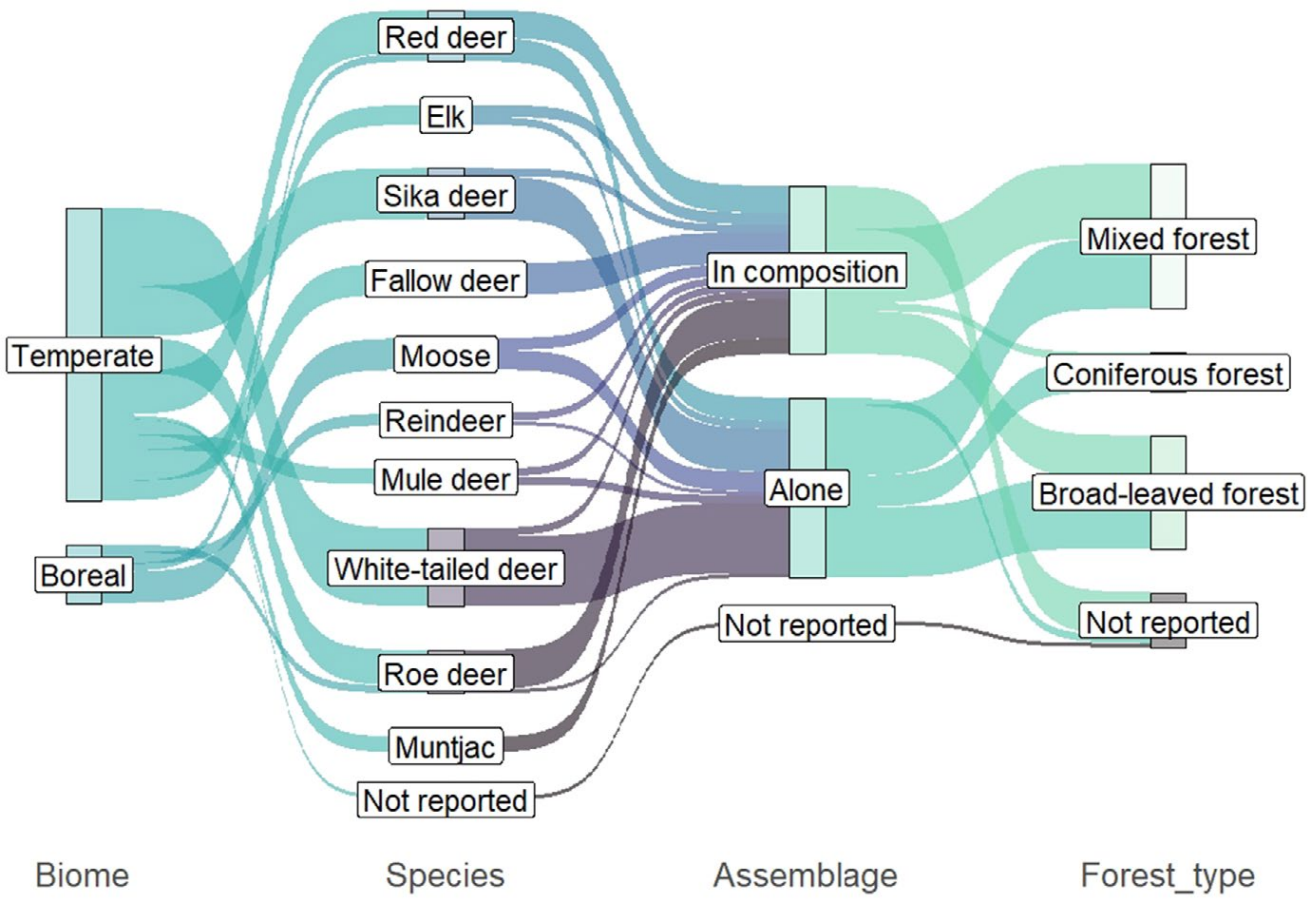
Sankey plot displaying the frequency of individual deer species studied and the respective distribution among biomes and forest types, as well as the frequency in which deer species were studied exclusively or in areas also inhabited by other deer species.

Overall, white‐tailed deer was the most commonly studied deer species (*n* = 20), followed by red deer and sika deer, which were studied in 13 studies each (Figure 4). Furthermore, white‐tailed deer and sika deer were almost always the only species present in their study areas, accounting for 90% and 84.6% of the studies on white‐tailed or sika deer, respectively. In contrast, roe deer and fallow deer are almost exclusively studied in combination with other deer species, while most other deer species were studied alone as well as together with other species. Reindeer is the least studied native deer species (*n* = 3), and one study from North America did not specify which deer species was assessed. In total, 18 studies assessed the effects of more than one deer species, for which the average number of deer species present was 2.4. Notably, one study assessed a system with a total five deer species, of which two were non‐native. When grouping deer species according to their respective feeding type, most studies report on the effects of browser only (*n* = 27), followed by studies with only intermediate feeders (*n* = 22), while both feeding types are present in 14 studies. Regarding deer species in relation to forest type, mule deer, moose and reindeer have only been studied in coniferous or mixed forest, while fallow deer, roe deer and white‐tailed deer were only studied in broad‐leaved or mixed forest. Sika deer, elk, and red deer were studied in all forest types, but red deer had the highest proportion of studies in coniferous forest compared to other deer species (30.7%).

### 3.3 Faunal Taxa Studied

**FIGURE 5:**
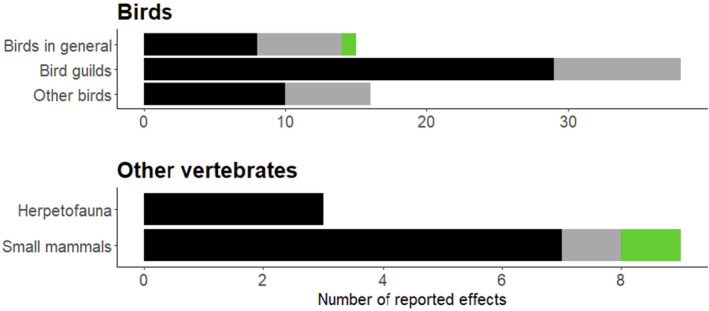
Reported effects for vertebrate taxa. Black bars indicate effects on faunal abundance, gray faunal species richness, and green faunal diversity. Effects for bird guilds include nesting guilds while feeding guilds and categories like “forest specialists” or “migrants” are reported under “Other birds.”

In total, 393 effects of deer on faunistic abundance (*n* = 283), species richness (*n* = 82), or diversity (*n* = 28) were reported in the analyzed studies. Of the effects found, 20.6% were on vertebrate taxa of which birds were the most intensively studied with 69 reported effects (Figure 5). For small mammals (rodents) and herpetofauna only nine and three effects were reported respectively, while for other vertebrate groups such as larger mammals or bats, no studies were found that met the criteria of our search. Additionally, 34 studies also assessed the composition of faunistic groups on the species level.

**FIGURE 6:**
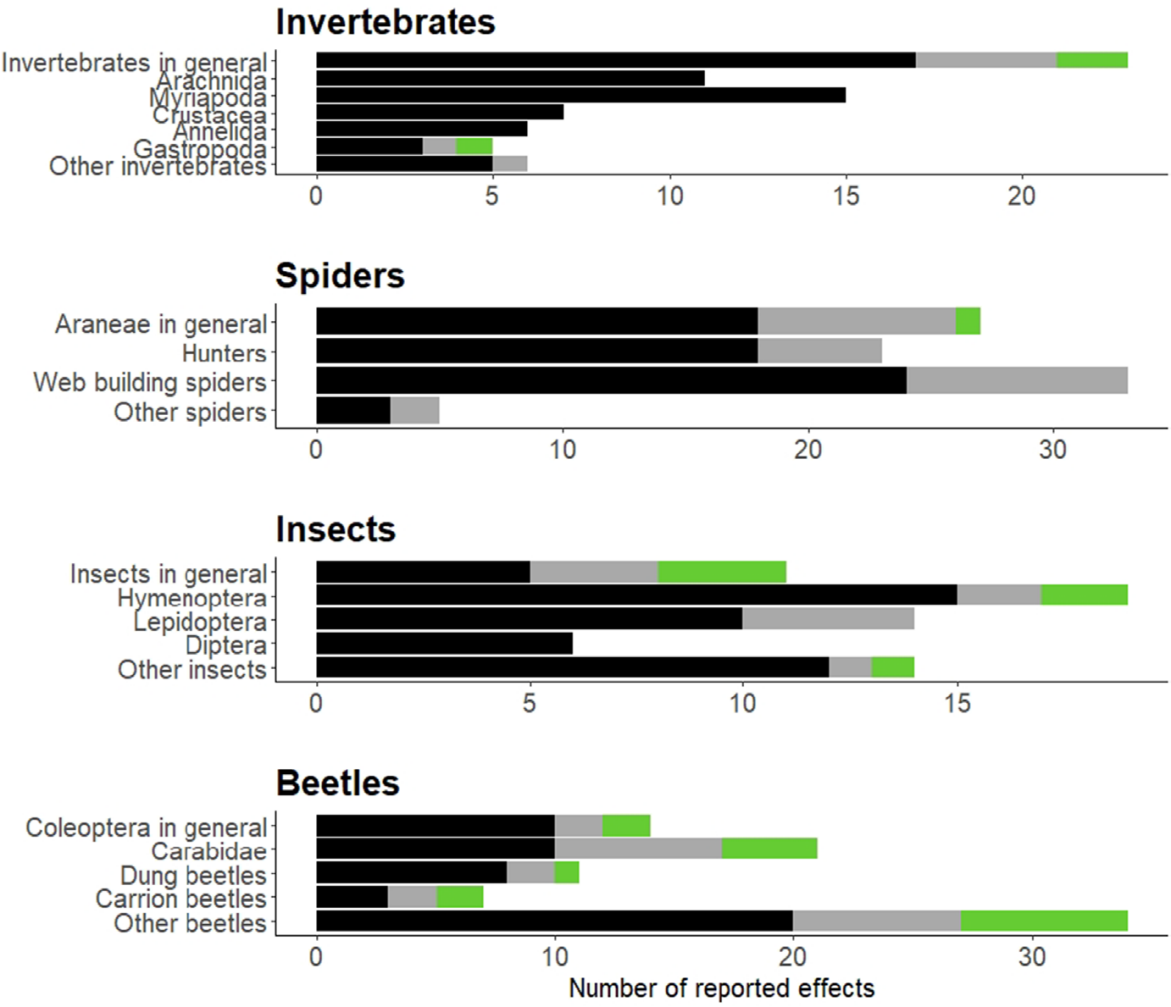
Reported effects for the influence on deer on invertebrate taxa. Black bars indicate effects on faunal abundance, gray faunal species richness, and green faunal diversity. The response “Invertebrates in general” also includes effects that were reported for arthropods. “Arachnida” does not include spiders as these are reported individually. The category of “Other invertebrates” includes groups like Collembola and Turbellaria. The category of “Web building spiders” also includes weavers. The category of “Other insects” includes orders like Hemiptera, Psocoptera, or Blattodea. The category of “Other beetles” mostly includes other beetle families such as Staphylinidae, Curculionidae, Cerambycidae, or Leiodidae.

The reported effects for deer on invertebrates varied widely at the taxonomic level (Figure 6). Spiders were the most studied non‐insect faunal taxon with 88 effects from 14 studies, most of which exclusively focused on spiders (e.g., Fuller et al. 2014; Saikkonen et al. 2019). After spiders, myriapods (*n* = 15) and other arachnids (*n* = 11) are the most studied non‐insect invertebrates. Among insects, beetles are the most studied taxa (*n* = 87). In particular, the family *Carabidae* was studied frequently (*n* = 21), even more than the other insect orders Hymenoptera (*n* = 19) and Lepidoptera (*n* = 14). In general, the focus on species diversity was higher in studies assessing insects with a total of 22 reported effects across all reported categories compared to only four diversity effects across all categories of non‐insect invertebrates.

**FIGURE 7:**
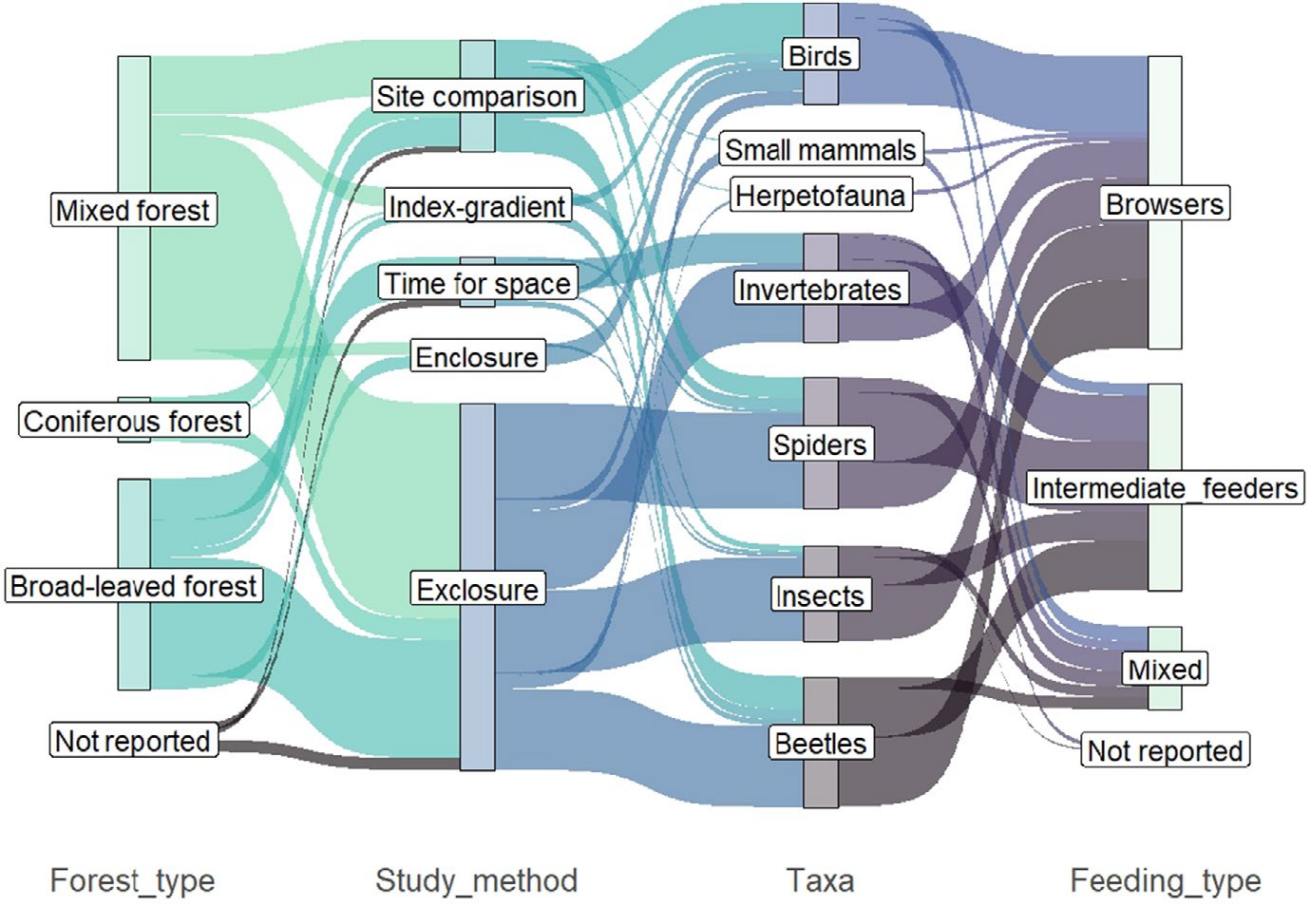
Sankey plot displaying the frequency of which faunal taxa were studied in relation to deer feeding type, methodological approach, and forest type. The method “index‐gradient” refers to studies using continuous indices, for example, from pellet counts as indirect variables representing deer abundance.

Most of the deer effects on faunistic groups have been reported in relation to both deer feeding types, browsers, and intermediate feeders (Table 2, Figure 7). However, the response of herpetofauna was only assessed in areas with white‐tailed deer and consequently only browsers. For small mammals on the other hand, browsers as well as intermediate feeders were present in all study areas. The response of birds to browsers—especially white‐tailed deer—was present in the selected studies, while there was only one study that assessed the effect of intermediate feeders only on birds. For most other faunistic groups, there is a relative balance between effects of browser and intermediate feeders. In general, however, for all insect groups only a few studies assess the effects of browsers and intermediate feeders together. Spiders and beetles were not studied in response to elk or mule deer; however, these deer species generally have not been focused on in general.

**TABLE 2:** Number of studies of different faunistic groups in relation to specific native deer species.

Deer species	Birds	S. Mammals	Herps	Invertebrates	Insects	Spiders	Beetles
Red deer	2 (1)	2 (2)	0	3 (2)	3 (1)	4 (3)	5 (1)
Elk	1 (1)	1 (1)	0	2 (1)	1 (0)	0	0
Sika deer	1 (1)	0	0	4 (0)	3 (1)	5 (2)	6 (1)
Fallow deer	4 (4)	2 (2)	0	2 (2)	1 (1)	1 (1)	1 (1)
Reindeer	0	0	0	1 (1)	0	1 (0)	1 (1)
Moose	1 (0)	1 (0)	0	2 (2)	3 (1)	1 (1)	4 (2)
Mule deer	1 (0)	1 (1)	0	1 (1)	1 (0)	0	0
W‐tailed deer	8 (1)	3 (1)	3 (0)	5 (0)	4 (0)	4 (0)	3 (0)
Roe deer	4 (4)	3 (3)	0	3 (3)	1 (1)	2 (2)	2 (1)
Feeding type							
Browsers	9	0	3	6	8	5	7
Intermediate feeders	1	0	0	6	6	7	10
Mixed	5	4	0	4	1	2	2

*Note:* The first number reports the number of studies, the number in parenthesis reports the number of studies in which the respective deer species occurred alongside other deer species. For example, eight studies report the effects of white‐tailed deer on birds, in seven of these studies white‐tailed deer was the only deer species present. The bottom part reports the number of studies in which the faunal group was assessed, either a system with only browsers, only intermediate feeders or a mix of both. S. Mammals: small mammals, Herps: herpetofauna, W‐tailed deer: white‐tailed deer.

### 3.4 Methods and Context of Encountered Studies

In total, 38 studies used fencing methods to study the effects of deer on fauna. Of these, 34 studies excluded deer via fences from certain forest areas, whereas 5 studies enclosed deer to simulate specific deer densities (Figure 7). Two of the enclosure studies used their methods to simulate four different levels of deer density, and 1 study applied a mix of exclosure and enclosure to create three levels of deer density (Iida, Soga, Hiura, et al. 2016). All remaining exclosure studies compared the complete exclosure of deer to an ambient deer density, resulting in two levels of deer abundance. The time between fencing and data collection ranged from 1 to 60 years. Of the remaining studies, five used index methods, correlating indices of deer abundance such as pellet counts with faunal data to assess the effect of deer. Two studies assessed fauna trends along a time series, during which also the deer density changed. The remaining 19 studies compared fauna between areas with different deer abundances.

In total, 42 studies (65.6%) reported on the deer densities in their respective study areas. However, the metrics often varied. Some studies reported a range of deer densities, some reported the average densities, while others reported maximum densities. Therefore, not all studies could be included in the calculating of an average deer density; however, based on 38 studies, the average deer density was 24.2 deer/km^2^. Reported densities ranged from 1.1 to 169 deer/km^2^ (Beckett et al. 2022). At least 26 studies assessed deer populations that locally reached up to 20 deer/km^2^, although the average density may have been lower. Of the remaining 22 studies that did not report densities, 7 reported alternative data such as the number of pellet counts or the hunting bag data, which can be indicators of deer density. However, 15 studies did not report any estimate of deer density or abundance. In total, 13 studies referred to their studied deer population as “overabundant” (or used similar terms such as overbrowsing or hyperabundant), while another 18 studies referred to the concept of overabundance while contextualizing their study. The context of conducted studies was not fully described in many studies; 67.2% of studies did not report whether the forest in the study area was managed or not. Of the remaining 21 studies, only two were conducted in unmanaged forests. Similarly, only 25% of studies (*n* = 16) reported whether deer were hunted in the study area. Specifically, the respective deer were hunted in nine cases. The presence or absence of natural predators was reported only in 12.5% of the studies, and natural predators were only present in three of these studies.

Almost all of the included studies that aimed to assess the effects of deer on forest fauna expected these effects to occur indirectly through deer effects on vegetation (96.9%). Consequently, 44 studies collected data on forest vegetation, in addition to faunal data. Another 13 studies assumed that the browsing on vegetation had cascading effects on characteristics of forest soil and leaf litter, and thus expected consequent changes in litter and soil dwelling fauna. Only two studies did not expect indirect effects through vegetation and assumed direct effects, for example, on dung beetles (Buse et al. 2021), or did not clarify why a faunal change was expected.

## 4 Discussion

### 4.1 State of Research and Knowledge Gaps

The effects of deer on faunistic groups have been intensively studied on all continents of the Northern Hemisphere over the last 2.5 decades, showing that the research community considers deer as a potentially powerful driver of diversity and composition of forest‐dwelling faunal communities. However, we found unever representation of study areas and study species, such as a lack of studies in Eastern Europe and Asia; a strong geographic bias toward studies from North America, especially on white‐tailed deer; and a bias toward literature on red and sika deer, while other deer species such as mule or fallow deer are underrepresented. Notably, in central Europe, few studies have been conducted in single deer species systems (see Cordeiro Pereira et al. 2024 for an exception). In particular, roe deer are widely distributed, and often occur sympatrically with other deer species, so that the specific study of roe deer and their effects on the ecosystem is difficult. In contrast, white‐tailed deer in eastern North America are often the only present deer species, resulting in many studies exclusively assessing the effect of white‐tailed deer. Thus, the comparative individual impacts of each species are hard to derive from the current literature. In addition, we found few studies assessing the effects of deer on fauna in boreal forest systems (*n* = 11) as well as pure coniferous forests (*n* = 9), which are likely connected patterns as pure coniferous forests are more common in the boreal zone.

Most of the studies that we included in this systematic map assess the effects of deer on either birds or invertebrates, most commonly beetles and spiders. While there are some studies assessing small mammals, the response of other vertebrates to deer was rarely assessed in the studies we found, although effects of deer on the communities of scavengers, predators and other herbivores (competitors) can be expected. For example, the importance of deer carcasses for scavengers is often described, but most of these studies were not retained in our literature search as scavengers are most often assessed at carcass sites, but not at the community level over larger spatial scales in relation to deer abundance (e.g., DeVault et al. 2011; Schwegmann, Storch, and Bhardwaj 2023; von Hoermann et al. 2021). We found one study assessing scavenger communities in response to deer density, but this study was conducted in open habitat and therefore not eligible for this review (Henden et al. 2014). Large predator communities on the other hand, are often composed of few or single species with low overall densities and large home ranges in boreal and temperate forests (Herfindal et al. 2005; Okarma et al. 1998). Therefore, assessing the predator community in response to varying deer densities, as was the premise of this study, is not practical, while studies assessing the effects on single species (e.g., Andrén and Liberg 2024) were generally excluded from this review. Similarly, studies assessing the effects on other large herbivores (e.g., bovids) were not included, as often few or only one species co‐occur alongside deer. Furthermore, while potential effects of deer on bats have been hypothesized (Palmer, Krueger, and Isbell 2019), we found no eligible studies, although one study assessed the potential of deer browsing (Barbaro et al. 2019).

Overall, we found far more reported effects of deer on faunal abundance than on diversity or species richness. For some taxa, such as birds or beetles, the species identification is relatively straightforward; for other groups of invertebrates, species identity and consequently measures of species richness and diversity are more difficult and laborious to obtain, which likely causes this pattern. While trends in faunal abundance can help us understand biotic interactions, a greater focus on diversity responses is needed for a more holistic understanding of deer impacts.

### 4.2 Missing Links for Synthesis

Synthesis of the available research on the effects deer on forest fauna is difficult due to a number of underlying factors. First, there is a lack of investigation of potential non‐linear effects of deer on fauna. This is the result of the frequent application of two‐level exclosure experiments, comparing the complete absence of deer inside a fenced‐off area to local deer density (Bergström and Edenius 2003). While this approach allows researchers to establish clear causality between the deer impact and the faunal response, it implies a linear relationship between deer and fauna. However, because deer also perform a number of ecosystem functions such as providing carrion and dung, as well as the maintenance of forest gaps (Buse et al. 2021; Feber 2001; Iida, Soga, and Koike 2016; Kuijper et al. 2009; Stiegler et al. 2020), the categorical assumption of linear (negative) relationships between deer and fauna is not always justified and mostly based on observed negative effects from ecosystems with very high deer abundance (Crystal‐Ornelas et al. 2021; Martin, Arcese, and Scheerder 2011; Wheatall, Nuttle, and Yerger 2013). An increasing number of studies find positive relationships between deer and fauna, indicating that deer–fauna interactions are more complex (Cordeiro Pereira et al. 2024; Iida, Soga, and Koike 2016; Saikkonen et al. 2019). Thus, it may be more likely that the relationships between deer and other fauna follow a “hump‐shaped curve,” in accordance with the Intermediate‐Disturbance Hypothesis (Connell 1978). Future research investigating deer–fauna relationships should account for the possibility of non‐linear relationships by including more than two levels of deer abundance in the analysis. This would also allow to calculate site specific abundance thresholds, at which deer start to affect forest fauna negatively, which are currently lacking in the literature.

Another aspect that limits our understanding of deer–fauna relationships is the fact that many studies focus specifically on “overabundant” deer populations. This can introduce a bias when attempting to synthesize the effects of deer on fauna, as overabundant deer populations have, by definition, have a negative impact on the respective ecosystem (Côté et al. 2004; McShea, Underwood, and Rappole 1997). This is possibly caused by a publication bias, as very high deer abundances might cause more significant effects on fauna, which are therefore easier to publish. In general, researchers need to be explicit about the abundance or density of deer in the study area to allow for comparisons between studies, which was not the case for all studies included in this systematic map.

Site productivity is another impactful confounding variable. Deer at a given density may have no measurable effect on the vegetation or fauna in a productive forest site, while the same density of deer may have a significant effect in nutrient‐poor study sites (Bergström and Edenius 2003; Côté et al. 2004; Hester et al. 2000). However, site‐productivity is not regularly reported in studies (Hester et al. 2000). The current literature may be biased by a strong study focus on nutrient‐poor sites, where effects of deer might be inherently stronger, as noted by Hester et al. (2000). Similarly, forage availability may mediate how strongly deer affect faunal communities; for example, deer density and browsing impact are not always linearly linked and forage availability can be a crucial confounder of the deer browsing effects on vegetation (Kamler et al. 2010; Schwegmann, Mörsdorf, et al. 2023; Wright et al. 2012). Thus, future studies must work toward measuring and reporting site‐productivity, possibly by reporting climatic conditions, soil types, and potentially productivity indices (Gale, Grigal, and Harding 1991). Finally, control of deer populations, through predation, hunting and forest management are underreported aspects in studies, despite their potentially confounding nature. Specifically, fear from hunting or natural predation affects deer behavior such as habitat use or temporal activity patterns which might lead to sub‐optimal resource use, in relation to its availability (Gerhardt et al. 2013; Kautz et al. 2022; Kuijper et al. 2013; Nopp‐Mayr, Reimoser, and Voelk 2011). This in turn, could influence how the forest vegetation is affected by browsing with potential consequences for other faunal taxa. In addition, while forest management itself is also a strong driver of forest‐dwelling faunal communities (Burrascano et al. 2023; Uhl, Schall, and Bässler 2024), it can also be expected that vegetation‐mediated effects of deer on fauna are exacerbated by forest management that reduces forage availability. Forest management for timber production generally shortens the overall life cycle of forests, specifically reducing the availability of early and very late successional stages, which are particularly rich in forage (Burney and Jacobs 2013; Dittrich et al. 2013; Hilmers et al. 2018). Additionally, silvicultural management practices such as clear‐cutting or single‐tree selection can strongly affect the type and amount of available forage for deer through canopy light‐transmittance (Vospernik and Reimoser 2008). Currently, this is difficult to test because few studies offer sufficient information on forest management. Specifically, it would be valuable to not only compare the effects of deer in managed and unmanaged forests, but also the confounding effects of different forest management strategies. More detailed reporting on these aspects, hunting, predators, deer density, site productivity, and forest management will allow for more comprehensive synthesis and meta‐analysis.

### 4.3 Road to Synthesis

To better describe the role of deer density on deer–fauna relationships, we suggest the establishment of enclosure experiments with multiple levels of simulated deer densities that allow researchers to assess potential non‐linear effects of deer on forest fauna along a deer density gradient. The main advantage of this approach is that the exact deer density and the potential responses can be causally attributed to changes in deer density, while avoiding density fluctuations. Replication of such experiments allow evaluation of the effect of different deer species, single versus multiple deer species, feeding type, system productivity, or forest type. However, there are drawbacks to consider, beyond the obvious high costs and maintenance effort. Specifically, for migratory deer species, such as reindeer, the application of enclosures cannot fully represent natural effects, even if density fluctuations are taken into account. Furthermore, the faunal taxa which can be assessed with this method depend on the size of the individual exclosures. Because multiple enclosures per experiment have to be established, this method requires a significant amount of comparable forest habitat. The larger the assessed faunal taxa are, the larger the enclosures need to be as well, which can be a practical challenge. While the effects of deer on vegetation can be assessed using ten‐by‐ten‐meter plots, much larger fences may be necessary for birds or other vertebrates. Therefore, it will be impossible to assess the response of larger competitors, predators, and scavengers through this method. If enclosures prove to be too laborious, an additional option would be to establish exclosure experiments along a gradient of known population densities, so that the potential effects on fauna can be studied along a density gradient. In this case, however, detailed account on potential confounding factors (e.g., site productivity) and precise estimates of the ambient deer population density are necessary. If absolute density estimates are impossible to generate, researchers could still aim to obtain continuous index data (e.g., from camera traps or pellet counts). These would allow to assess potential non‐linear effects of deer on fauna and even without fencing methods can be used to describe non‐linear cooccurrence patterns of deer and fauna (Cordeiro Pereira et al. 2024; Gobbi et al. 2018; Takada et al. 2008).

Different approaches are needed to assess effects of deer on larger species. For example, by assessing population trends of deer and populations of other faunal species of interest over longer time periods so that potential relationships can be identified. It would also be important to assess how comparable different deer species are in their effects on forest communities. While we expect substantial differences between browsers and intermediate feeders, it would be valuable to know how comparable, for example, the effects of white‐tailed deer and roe deer on forest fauna are. Overall, we believe that multi‐level enclosure and large‐scale assessments of deer and fauna population trends, can contribute to our understanding of deer effects on forest fauna and allow for comparisons among different species.

## 5 Conclusion

Our systematic map shows the current availability of studies assessing the effects of native deer on forest faunal communities. Overall, we found a variety of different effects of deer on forest fauna within the 64 included studies. However, there are some research gaps with respect to geographic location, deer species and specific faunistic groups. Although we did not assess the actual response of fauna to different deer abundances, the literature reports both positive and negative effects, incentivizing proper synthesis and meta‐analysis. Currently, this is difficult, because information contextualizing the individual studies, especially deer densities, are often lacking. It is imperative that studies report information about their study areas, including forest type and deer densities to allow for comparisons between studies and across different forest contexts. In addition, studies should move away from strict exclosure experiments, that do not allow the assessment of the faunal response to a deer density gradient. While very high deer densities have been found to negatively affect ecosystems, deer also provide ecosystem functions, suggesting that deer–‐fauna relationships are non‐linear. Therefore, we propose the application of enclosure experiments simulating multiple deer densities along a density gradient in the future, which would allow better comparison and synthesis between studies. Assessing the effects and differences between individual deer species as well as feeding types may support a more differentiated understanding of deer as biotic drivers, and consequently allow for more adapted deer management for the sake of forest biodiversity conservation.

## Author Contributions

**Sebastian Schwegmann:** conceptualization (lead), investigation (lead), methodology (lead), visualization (lead), writing – original draft (lead), writing – review and editing (equal). **Manisha Bhardwaj:** conceptualization (supporting), investigation (supporting), supervision (equal), visualization (supporting), writing – original draft (supporting), writing – review and editing (equal). **Ilse Storch:** conceptualization (supporting), funding acquisition (lead), project administration (lead), supervision (equal), writing – original draft (supporting), writing – review and editing (equal).

## Conflicts of Interest

The authors declare no conflicts of interest.

## Supporting information


Appendix S1

## Data Availability

The data supporting this systematic map are derived from published literature and do not constitute original data collected by the authors.
