# Supplementary material for: The Influence of Native Deer on Forest Fauna—A Systematic Map
Source: Ecol Evol. 2024 Dec 23;14(12):e70696. doi: 10.1002/ece3.70696 (PMC11664241; doi:10.1002/ece3.70696)
Supplement: Supplementary file 1 — Appendix S1 [file ECE3-14-e70696-s001.docx]

# Appendix A: Search string used in web of science

(ungulate OR deer OR cervidae OR cervid OR brows* OR cervus OR elk OR moose OR “rangifer tarandus” OR capreolus OR “reeves muntjac” OR “muntiacus reevesi” OR stag OR odocoileus OR “dama dama” OR “alces alces” ) AND (biodiversity OR fauna OR animal OR bird OR avifauna OR avian OR aves OR mice OR mouse OR shrew OR mammal OR vole OR dormouse OR dormice OR rodent OR squirrel OR eulipotyphla OR insectivore OR hare OR lagomorpha OR bat OR chiroptera OR herpetofauna OR snake OR salamander OR amphibian OR reptile OR frog OR toad OR newt OR lizard OR invertebrate OR insect OR arthropod OR beetle OR butterfly OR butterflies OR heteroptera OR homoptera OR hemiptera OR odonata OR orthoptera OR lepidoptera OR coleoptera OR hymenoptera OR diptera OR dermaptera OR siphonaptera OR psocoptera OR phthiraptera OR spiders OR araneae OR ticks OR ixodida OR collembola OR springtails OR gastropod OR slug OR snail OR miner OR soil OR annelida OR earthworm OR lumbricidae ) AND (assemblage OR diversity OR biodiversity OR composition OR community OR abundance OR species OR richness OR population) AND (forest* OR woodland OR wood* OR plantation OR silvi* OR timber OR coppice OR parkland OR stand) NOT (“key deer” OR “barking deer”)
